# *O*-GlcNAcylation and the Metabolic Shift in High-Proliferating Cells: All the Evidence Suggests that Sugars Dictate the Flux of Lipid Biogenesis in Tumor Processes

**DOI:** 10.3389/fonc.2016.00006

**Published:** 2016-01-22

**Authors:** Steffi F. Baldini, Tony Lefebvre

**Affiliations:** ^1^University Lille, CNRS, UMR 8576, UGSF, Unité de Glycobiologie Structurale et Fonctionnelle, Lille, France

**Keywords:** FAS, *O*-GlcNAcylation, OGT, cancer, cell proliferation

## Abstract

Cancer cells are characterized by their high capability to proliferate. This imposes an accelerated biosynthesis of membrane compounds to respond to the need for increasing the membrane surface of dividing cells and remodeling the structure of lipid microdomains. Recently, attention has been paid to the upregulation of *O*-GlcNAcylation processes observed in cancer cells. Although *O*-GlcNAcylation of lipogenic transcriptional regulators is described in the literature (e.g., FXR, LXR, ChREBP), little is known about the regulation of the enzymes that drive lipogenesis: acetyl co-enzyme A carboxylase and fatty acid synthase (FAS). The expression and catalytic activity of both FAS and O-GlcNAc transferase (OGT) are high in cancer cells but the reciprocal regulation of the two enzymes remains unexplored. In this perspective, we collected data linking FAS and OGT and, in so doing, pave the way for the exploration of the intricate functions of these two actors that play a central role in tumor growth.

Fatty acids are not only a form of energy storage but are also major components of biological membranes that delimit cells and organelles. They also govern essential biological functions, which include signal transduction and cell signaling, by forming exchange platforms between extracellular and intracellular media, in which signaling proteins are anchored.

Fatty acids are provided either by diet or *de novo* synthesis from carbohydrates. This metabolic pathway, called lipogenesis, is governed by fatty acid synthase (FAS), an enzyme upregulated in cancer. The activity and expression of O-GlcNAc transferase (OGT) that catalyzes *O*-GlcNAcylation processes in a nutrient-dependent manner are also increased in tumor cells. Consequently, increases in fatty acids production and *O*-GlcNAcylation levels are observed in cancer. Both enzymes indirectly use the same substrate, glucose, whose flux is potentiated in cancer cells. Moreover, FAS and a fraction of OGT are in close proximity at the plasma membrane. Therefore, a cross-regulation between FAS and OGT is likely to exist.

## FAS and OGT are Upregulated in Cancer

**Figure 1:**
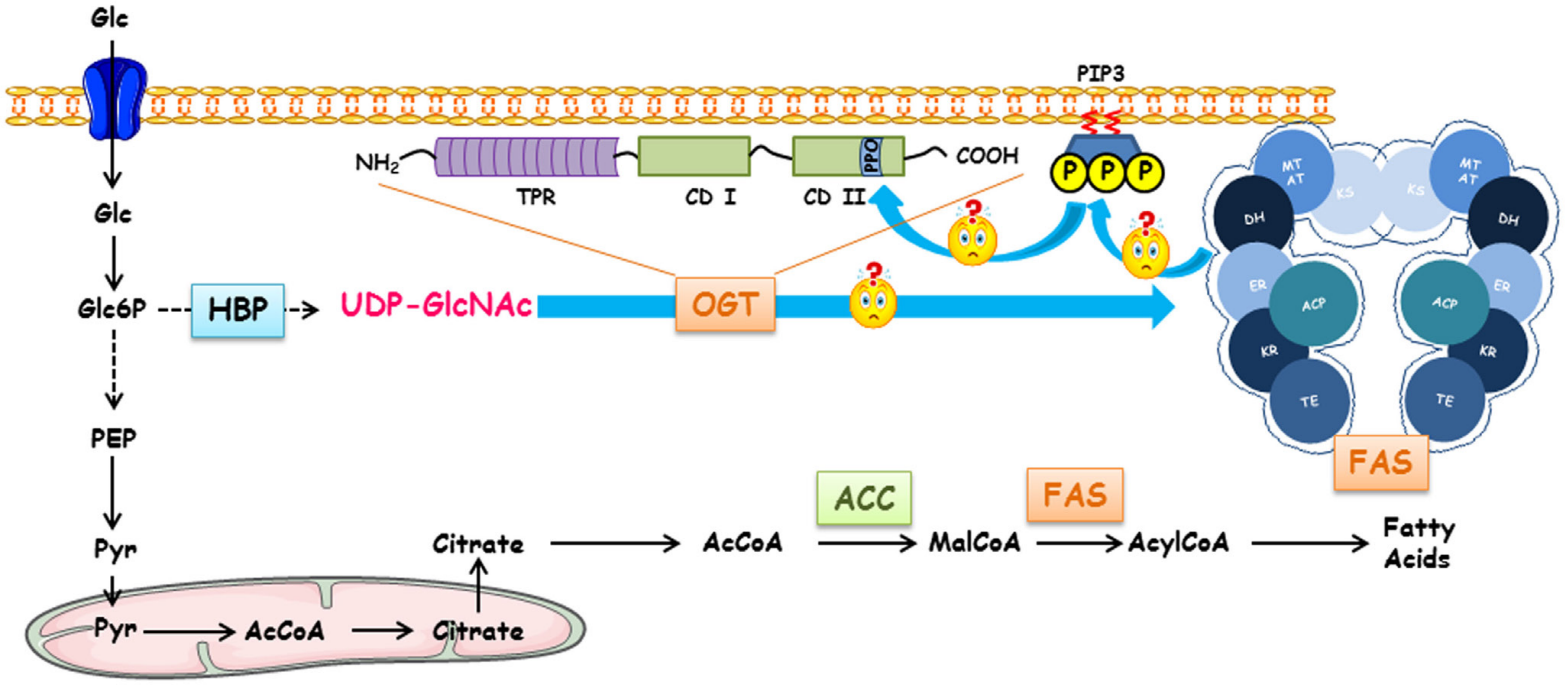
**Regulation of fatty acid synthase by *O*-GlcNAcylation, *Terra incognita***. FAS expression and *O*-GlcNAcylation levels are dependent on glucose concentrations. Following its entry into the cell, glucose can engage in different metabolic pathways depending on the cell type and the physiological context (glycolysis, glycogenogenesis, pentose phosphate pathway, etc.). HBP converts a fraction of glucose into UDP-GlcNAc, the donor for *O*-GlcNAcylation processes. Either for storing energy or for the biosynthesis of membranes of proliferating cells, a large pool of glucose can be used for making fatty acids of which FAS (fatty acid synthase) is a key enzyme. FAS forms a head-to-head X-shaped homodimer. Each 273 kDa-subunit of FAS is an assembly line of fatty acids endowed with seven different catalytic activities. First, the acetyl moiety provided by acetyl-CoA is transferred to the thiol group of the phosphopantetheine part of ACP (acyl carrier protein) that transports the growing fatty acid chain from one catalytic domain to another via the acetyltransferase (AT); it is presented to ketoacylsynthase (KS). Then, malonyl transferase (MT) transfers the malonyl moiety from malonyl-CoA to ACP, and KS condensates the malonyl and acetyl groups. The β-ketoacyl-ACP is modified by a succession of three reactions. Ketoreductase (KR) reduces β-ketoacyl to a β-hydroxyl intermediate; a dehydratase (DH) produces a β-enoyl-ACP, which is reduced by the enoyl reductase (ER) to supply a saturated acyl chain elongated by a two-carbon unit. This product is the substrate for the condensation with another malonyl-ACP in the next sequence of elongation. This cycle is repeated until a C16:0 is reached; the end product is finally released by the thioesterase activity (TE). Since there is a close Relationship between intracellular glucose, *O*-GlcNAcylation levels, and activation of glucido-lipidic metabolism, a link between OGT, O-GlcNAcylation level, expression, and activity of FAS should exist. In cancer cells, the increased expression of OGT might lead to an increase in FAS expression. FAS participates in the production of phosphoinositides found in lipid rafts; these lipid microdomains can recruit OGT in the close proximity of FAS increasing its activity and, thus, to an expansion of membrane surface necessary for cell proliferation.

In physiological conditions, circulating lipids supply most human tissues with fatty acids. In striking contrast, high-proliferating cancer cells use increased fatty acid synthesis to support their rate of division (1, 2). *De novo* lipogenesis converts glucose to fatty acids. First, glucose is metabolized into pyruvate, the end product of glycolysis that in turn enters the mitochondria to be activated into acetyl-CoA (Figure 1). The Lardy cycle enables the release of acetyl-CoA in the cytosol where lipogenesis takes place. Acetyl-CoA is carboxylated by Acetyl-CoA Carboxylase (ACC) to form malonyl-CoA. Malonyl-CoA and acetyl-CoA are the substrates of FAS that forms the final product, palmitoyl-CoA (C16:0). Longer fatty acids (e.g., stearic acid, C18:0) are produced by elongases, and mono-insaturated palmitoleoyl-CoA (C16:1) and oleyl-CoA (C18:1) are produced by SCD-1 (Stearoyl-CoA desaturase 1). ACC, FAS, and SCD-1 are the main enzymes that control lipogenesis (3–6).

Overexpression of FAS and enhancement of its activity represent one of the most frequent phenotypic alterations in tumor cells. FAS is overexpressed in several human carcinomas, such as breast (7), colon (8), esophageal (9), lung (10), melanoma (11), ovarian (12), pancreatic (13), prostate (14), and stomach (15) cancers. Injection of transgenic mice with prostate cancer cells overexpressing FAS leads to the development of adenocarcinomas (16). On the contrary, the FAS inhibitor C75 drives apoptosis in human breast cancer cells (17, 18). ACC is overexpressed in breast (19) and prostate cancers, and blockade of its expression induces growth inhibition of cancer cells (20). Additionally, an elevated SCD-1 expression results in cancer cell proliferation (21), whereas its decrease results in cell death (22).

Long-chain saturated fatty acids (LCSFA) are crucial for the building of lipid microdomains, or detergent-resistant membranes (DRMs). DRMs ensure the correct localization and activation of receptors that potentiate signaling pathway activation and, consequently, cell proliferation (1, 23). Moreover, the switch from polyunsaturated to saturated and mono-unsaturated fatty acids in the composition of their membranes endows cancer cells with a better resistance to lipid peroxidation and, therefore, to oxidative stress-induced cell death (24). Furthermore, the increase in membrane density and compaction limits drug entry, rendering tumor cells more resistant to therapy. Finally, fatty acids contribute to the production of secondary messengers, such as phosphatidylinositol-3,4,5-triphosphate (PIP3) and lysophosphatic acid (LPA), that activate, respectively, the mitogenic PI3-kinase/Akt pathway (25) and G protein coupled-receptors that promote cancer aggressiveness (26). Consequently, fatty acids confer survival, resistance to damage, and aggressiveness on cancer cells.

Glucose consumption is vital for most living cells. In normal cells, glucose is catabolized to pyruvate through glycolysis (Figure 1). Then, pyruvate is converted to acetyl-CoA and oxidized to carbon dioxide via the mitochondrial tricarboxylic acid (TCA) cycle for maximal energy production. Cancer cells exhibit a deregulated glucido-lipidic metabolism. Indeed, cancer cells import glucose at a higher rate than non-cancer cells and prefer to use glycolytic metabolism instead of oxidative phosphorylation even in the presence of oxygen. This choice enables most cancer cells to produce the elementary building-bricks they need to divide (amino-acids, nucleotides, fatty acids,… etc.). Consequently, glycolytic enzymes and glucose transporters are upregulated. This adaptive metabolic shift is called the “Warburg effect” (27).

Interestingly, 2–3% of glucose coming into the cell enters the HBP (hexosamine biosynthesis pathway) whose end product is UDP-GlcNAc. This nucleotide sugar is at the crossroads of many metabolic pathways: carbohydrates, glutamine, glucogenic and ketogenic amino-acids, nucleotides, and fatty acids. UDP-GlcNAc is a substrate for almost all glycosylations, including *O*-GlcNAcylation (28, 29). *O*-GlcNAcylation is a highly dynamic PTM (posttranslational modification) controlled by two antagonistic enzymes: OGT transfers the GlcNAc group onto serine or threonine residues of nucleocytoplasmic (30) and mitochondrial (31) proteins, and *O*-GlcNAcase removes the sugar (32). Thus, *O*-GlcNAcylation regulates diverse biological processes in a nutrient-dependent manner. *O*-GlcNAcylation occurs on several hundred proteins, which are mainly phosphoproteins. Accordingly, *O*-GlcNAcylation and phosphorylation can antagonize each other, at the same site or at an adjacent one, or act in concert (33). Moreover, a deregulation of *O*-GlcNAcylation homeostasis leads to disorders, such as diabetes, cancer, neurological, or cardiovascular diseases (34). Since, cancer cells accelerate the uptake of glucose and glutamine, an increase in HBP flux and aberrant *O*-GlcNAcylation contents result. Elevation of *O*-GlcNAcylation levels is observed in several kinds of cancers, such as breast (35), lung (36), colorectal (37), liver (38), bladder (39), prostate (40), chronic lymphocytic leukemia (CCL) (41), and pancreatic (42) cancers, suggesting that hyper-*O*-GlcNAcylation precedes or accompanies tumor emergence and contributes to cell transformation. The *O*-GlcNAcylation elevation observed in cancer cells seems also to be caused by an increased OGT expression. Indeed, OGT is overexpressed in breast (35), prostate (40), colorectal (36, 43), and pancreatic cancer cells (42). Interestingly, the inhibition of OGT in breast cancer cells induces a reduction in cell proliferation, invasion, and migration, and a decrease in tumor growth (35, 44).

An upregulation of FAS and OGT, and consequently an elevated production of fatty acids combined with an increase in the *O*-GlcNAcylation level are observed in cancer. Both enzymes indirectly use the same substrate, glucose, and glutamine for OGT, the fluxes of which are accelerated in tumor cells (Figure 1). Moreover, FAS and a fraction of OGT are in close proximity at the plasma membrane. Therefore, we postulate that FAS favors the interaction of OGT with phosphoinositides via its PPO (PIP-binding activity of OGT) domain and in turn OGT controls the expression and catalytic activity of FAS (Figure 1).

## Future Directions

**Figure 2:**
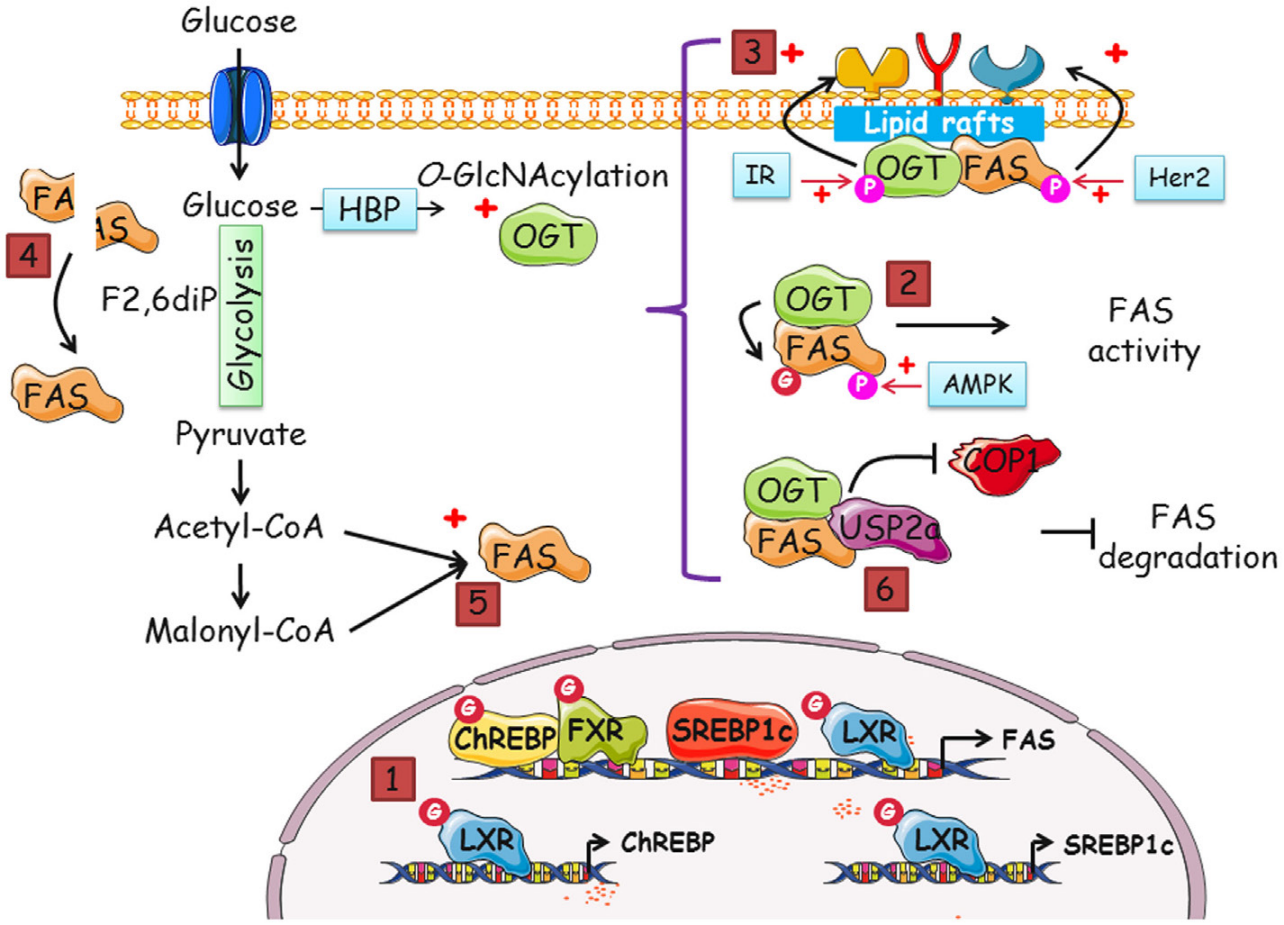
**Many putative pathways exist to regulate FAS by OGT and *O*-GlcNAcylation, and vice versa**. Increased-*O*-GlcNAcylation and FAS overexpression are two features of cancer cells. In this perspective, we gathered data that suggest that a dialog between OGT and FAS potentiates cell proliferation. The different modes by which FAS and OGT might interact are the following: (1) indirectly, FAS mRNA level is regulated by *O*-GlcNAcylation. The transcription of FAS is coordinated by four transcription factors, ChREBP, SREBP1c, FXR, and LXR. SREBP1c and ChREBP are themselves regulated by LXR in an *O*-GlcNAcylation-dependent manner. The activity of ChREBP, FXR, and LXR is directly linked to their *O*-GlcNAcylation status. (2) There are few descriptions of the modification of FAS by phosphorylation but a complex interplay between *O*-GlcNAcylation and phosphorylation cannot be discarded. (3) In cancer cells, both FAS and OGT are upregulated. It is also known that the enzymes are resident in lipid rafts, platforms of signal transduction and cell signaling. Thus, FAS and OGT might be interacting partners in lipid microdomains. FAS may contribute to the interaction of OGT with a specific subset of fatty acids of lipid microdomains, especially phosphoinositides, and, in turn, OGT may upregulate FAS activity. Consequently, receptor tyrosine kinases should cluster and increase the activation of signaling pathways and cell proliferation. (4) FAS homodimer assembly is F2,6BP dependent. F2,6BP also controls the oligomerization of PFK-1, the rate-limiting enzyme of glycolysis (63). *O*-GlcNAcylation of PFK-1 at S529 impinges on the binding site of F2,6BP and negatively regulates its activity. By contrast, we speculate whether *O*-GlcNAcylation favors FAS dimerization and activity. (5) The increased-flux of glucose uptake observed in cancer cells enhances the production of acetyl-CoA and malonyl-CoA, the substrates of FAS, and UDP-GlcNAc (in parallel with the accelerated-flux of glutamine in tumor cells), the donor of GlcNAc for the *O*-GlcNAcylation processes. It can be envisioned that one or several catalytic activities of FAS are directly under the control of OGT through their *O*-GlcNAcylation. Therefore, FAS might be controlled by glucose in two distinct ways: by enhancing the availability of substrates and by the posttranslational modification by *O*-GlcNAc moieties. (6) The *O*-GlcNAcylation of FAS may potentiate the interaction with the deubiquitinase USP2a and/or destabilize its interaction with the E3-ubiquitin ligase COP1, rendering the enzyme less sensitive to ubiquitin-dependent degradation.

Glucose and insulin control the expression of FAS at the transcriptional level through the activity of four transcription factors: carbohydrate-responsive element-binding protein (ChREBP), liver X receptors (LXR), farnesoid X receptor (FXR), and sterol regulatory element-binding proteins (SREBP) (Figure 2, *pathway 1*). ChREBP is a transcription factor that mediates glucose-induced gene expression, including those encoding the glycolytic enzyme L-pyruvate kinase (L-PK) and lipogenic enzymes, ACC, and FAS. Glucose activates ChREBP by controlling its nuclear import (45) and ChREBP plays an essential role in the regulation of energy metabolism. A deregulation of ChREBP activity or its expression is involved in metabolic diseases, such as hepatic steatosis (46). Moreover, a deregulation of ChREBP is described in colorectal cancer and hepatoblastoma. A suppression of ChREBP decreases aerobic glycolysis, *de novo* lipogenesis, and nucleotide biosynthesis (47). Since cancer cells overconsume glucose and increase the biosynthesis of fatty acids, the role of ChREBP as a key-mediator of glucose-induced lipogenic gene expression was explored in the context of tumor metabolism (48). In response to high glucose influx as observed in cancer cells, ChREBP is activated in two distinct ways. First, controversial studies reported that Xu5P and Glc6P, two glucose metabolites, promote the translocation of ChREBP into the nucleus and binding to response elements (49). Second, ChREBP physically interacts with OGT and is *O*-GlcNAcylated in liver cells (50). Inhibition of OGA, or overexpression of OGT, significantly increases the transcriptional activity of ChREBP in response to high glucose. OGA-treated db/db mice display a decrease in FAS expression and escape hepatic steatosis in correlation with a downregulation of the *O*-GlcNAcylation of ChREBP (50, 51). LXRs belong to the superfamily of nuclear hormone receptors activated by the natural ligands oxysterols (52). LXRs bind to LXR-responsive elements (LXRE) in cooperation with RXR (9-*cis* retinoic acid receptor) and activate FAS transcription. LXRs either directly bind to the promoter of FAS, or indirectly increases the expression of FAS by upregulating the transcription of ChREBPα, the short isoform ChREBPβ and SREBP1c (53, 54). LXRs are *O*-GlcNAc-modified (55, 56) and *O*-GlcNAcylation enhances their ability to regulate FAS expression. Furthermore, *O*-GlcNAcylated LXRs have increased capability to activate the transcription of SREBP1c and ChREBP, which reinforces the expression of FAS. Regarding SREBPs less is known about their potential regulation by *O*-GlcNAcylation. Of the three isoforms documented, SREBP1a, SREBP1c and SREBP2 (57), only SREBP1c is involved in the expression of FAS (58). No studies have focused on the regulation of SREBP by *O*-GlcNAcylation, while it is thought that GFAT-1, the rate-limiting enzyme of HBP, interferes with the activity of SREBP1 and FAS (59). Interestingly, an FXR response element is found in the promoter region of FAS. FXR is another member of the nuclear receptor superfamily and is activated by bile acids, chenodeoxycholic acid being the most potent. Unlike other transcription factors, FXR downregulates FAS expression (60). It was recently shown that FXR and OGT interact (61). FXR is *O*-GlcNAcylated and this modification enhances FXR transcriptional activity and stability. Interestingly, FXR interacts with ChREBP through its ATF-1 domain, which also bears the *O*-GlcNAcylation site (61, 62). However, the biological relevance of FXR *O*-GlcNAcylation in the context of its interaction with ChREBP remains to be elucidated. Transcriptional processes may account for a large part of the upregulation of FAS in cancer. What we do not know is whether, and if so, how, an elevation of the *O*-GlcNAcylation status of ChREBP, SREBP, and LXR, all driving FAS transcription, mediates the lipogenic enzyme overexpression in cancer cells, and, on the opposite, if *O*-GlcNAcylation of FXR reduces its expression. Further studies are necessary to highlight this intriguing problem.

*O*-GlcNAcylation and phosphorylation are two PTMs occurring on serine and threonine residues of nucleocytoplasmic proteins. They not only can compete at the same or at adjacent sites of the same protein in a reciprocal manner, but can also act synergistically: treatment of neuroblastoma cells with the wide range phosphatase inhibitor okadaic acid reduces *O*-GlcNAcylation (64), while inhibition of GSK3β increases *O*-GlcNAcylation of heat shock and cytoskeletal proteins but decreases the *O*-GlcNAcylation of other proteins such as transcription factors (65). These observations prove that the interplay between phosphorylation and *O*-GlcNAcylation is complex and should result from diverse complexes, including OGT, OGA, kinases, and phosphatases (66). Little is known about the regulation of FAS by PTMs. FAS is phosphorylated by AMP-activated protein kinase (AMPK) in 3T3-L1 cells and the use of AMPK activators reduces FAS activity without affecting its expression level (67). An upregulation of FAS by occupation of the AMPK phosphorylation site by *O*-GlcNAc is hypothetical, but deserves to be explored (Figure 2, *pathway 2*). In addition, OGT is a substrate of AMPK (at T444) and reciprocally, AMPK is *O*-GlcNAcylated on at least two of its subunits (68). The potential occurrence of a ternary complex FAS–OGT–AMPK may explain how the lipogenic enzyme is regulated by phosphorylation and *O*-GlcNAcylation either directly, involving modification by AMPK-directed phosphorylation and/or *O*-GlcNAcylation, or indirectly, with phosphorylation by AMPK through a preliminary *O*-GlcNAcylation of the kinase or *O*-GlcNAcylation by OGT following a preliminary modification of the glycosyltransferase by AMPK (pT444).

O-GlcNAc transferase and FAS might reciprocally regulate each other in lipid microdomains and *O*-GlcNAcylation might interfere with FAS subcellular localization. It was demonstrated that FAS is tyrosine-phosphorylated when in a complex with HER-2 (human epidermal growth factor receptor-2) in breast cancer cells (69). Phosphorylation of FAS by HER-2 leads to an increase in FAS catalytic activity. OGT is also tyrosine-phosphorylated and it was proposed that the insulin receptor is responsible for this modification (70). Moreover, OGT interacts with the plasma membrane via the PPO domain (71) and more particularly with DRMs in response to insulin (72). DRMs, also known as lipid microdomains or lipid rafts, are membrane domains enriched with cholesterol, sphingolipids, and gangliosides that concentrate modulators of signal transduction, trafficking, and cell signaling. Lipid rafts are fundamental for cell adhesion, migration, and proliferation in physiological conditions and in cancer (73). FAS controls the production of phospholipids found in DRMs of cancer cells (1) and a subset of FAS itself is resident in these rafts (74). Consequently, in response to insulin or to any other mitogenic signal, FAS might be activated by tyrosine-phosphorylation. In turn, raft-specific phospholipids and more precisely phosphoinositides might be produced, offering an interaction surface for OGT (Figure 2, *pathway 3*). FAS and OGT might physically interact: OGT may modify FAS and signaling components to upregulate their activities. This interaction could induce an increase in FAS activity in lipid microdomains and allow the formation of a larger cluster of signaling receptors and a more significant activation of signaling pathways, leading to an increase in cell proliferation, contributing to the development of cancer. It would be of interest to determine whether a synergistic effect between FAS and OGT exists in lipid rafts, accentuating their respective activities. This hypothesis is supported by reports showing that, in *Drosophila* (75), HepG2 cells (72), MCF7 cells (76), and recently in human gastric cancer cells (77), OGT activity is crucial for activating mitogen signaling pathways.

Another question remains open: does *O*-GlcNAcylation regulate FAS catalytic activity? FAS is only enzymatically active in the form of a dimer (78), the dissociation of the enzyme into monomers rendering it inactive (79). The assembly of the two subunits is promoted by a high concentration of fructose-2,6-bisphosphate (F2,6BP), a metabolic cue. Therefore, since in cancer cells glucose uptake is increased, the rate of production of F2,6BP is enhanced, promoting FAS assembly and activity. A parallel can be drawn with Phosphofructokinase-1 (PFK-1) whose allosteric activity is positively regulated by F2,6BP. *O*-GlcNAcylation of PFK-1 blocks the interaction with F2,6BP preventing the oligomerization of the enzyme and the flux of glucose is rerouted to the pentose phosphate pathway (63). Conversely, a regulation of FAS activity by promoting its dimerization by *O*-GlcNAcylation could be envisioned (Figure 2, *pathway 4*). *O*-GlcNAcylation can impact the function of FAS by directly modifying residues crucial for one or several of its seven catalytic activities. In our hands, *in silico* studies of the 3D structure of FAS have indicated a potential *O*-GlcNAcylation site in close proximity to the ketoacylsynthase (KS) active site (Figure 2, *pathway 5*). As discussed above, these studies must be pushed forward by identifying the *O*-GlcNAcylation sites through mass spectrometry.

Lastly, *O*-GlcNAcylation might regulate the fate of FAS. FAS is targeted for degradation by interacting with the E3-ubiquitin ligase p38-phosphorylated COP1 and the adapter protein Src-homology 2 (SH2) domain-containing tyrosine phosphatase Shp2 (80). To escape the ubiquitination pathway, FAS interacts with the ubiquitin-specific protease-2a USP2a, a member of the DUB (deubiquitinating enzyme) family. By binding to FAS, USP2a increases the half-life of the enzyme in prostate cancer and, therefore, plays a prominent role in cancer progression (81). In addition, USP2a overexpression protects human prostate cancer from apoptosis (82). Altogether, these observations indicate that USP2a and FAS contribute to tumorigenesis. Like USP2a, OGT is also protective against protein degradation for a subset of proteins [for review, see Ref. (83)] among which β-catenin (43) and PGC-1α (84). *O*-GlcNAcylation of β-catenin at T41 prevents the phosphorylation of the D-box (43) and potentially the subsequent recruitment of the E3-ubiquitin ligase β-TrCp. The host cell factor C1 (HCF-1) recruits OGT to *O*-GlcNAcylate PGC-1α (84); the *O*-GlcNAcylation facilitates the recruitment of BAP1 to deubiquitinate PGC-1α, thereby stabilizing its expression level. Nevertheless, unlike β-catenin, the sequential events that decide the fate of FAS are poorly understood; it is not known, for example, whether phosphorylation of FAS promotes its ubiquitination. Also, since FAS is not a short half-life protein, it is unlikely that a PEST sequence or a D-box regulates the proteasomal degradation of the protein. Accordingly, it might be proposed that OGT interacts with FAS either to promote the recruitment of USP2a or to prevent ubiquitination by COP1, both effects leading to increased-FAS half-life (Figure 2, *pathway 6*).

## Conclusion

Cancer cells are characterized by their high capacity for division. To supply their need for carbon elements, these cells increase the uptake of glucose and move from oxidative to glycolytic metabolism. This metabolic shift leads to the overexpression of lipogenic enzymes, especially FAS. In addition, through the increased flux of HBP, cancer cells display higher levels of OGT and, therefore, of *O*-GlcNAcylation. Here, we have attempted to compile potential cross-regulations between FAS and OGT. We have identified pathways to explore that may highlight how the lipogenic enzyme could be finely tuned by *O*-GlcNAcylation.

First, upregulating glycolysis impacts the production of the allosteric activator F2,6BP. Checking whether this metabolic cue controls the homodimerization of FAS in an *O*-GlcNAc-dependent manner should be of great interest considering its impact on the oligomerization of another pivotal metabolic enzyme, PFK-1. However, at this time, no information about the *O*-GlcNAc modification of FAS is available. This lack of data may result from the high molecular weight of the enzyme: analyzing the status of *O*-GlcNAcylation and, what is more, mapping sites of modification on a protein containing nearly 3000 amino-acids is challenging. If such a modification of FAS occurs, the consequent regulation of one or more of its catalytic activities is conceivable.

Regulation of FAS expression is tightly correlated to its mRNA level. Transcription of FAS is principally under the control of glucose and insulin. Glucose activates ChREBP that in turn induces FAS transcription in cooperation with insulin-activated SREBP1c. LXR also increases FAS expression by binding to the promoters of FAS and SREBP1c. ChREBP, SREBP1c, and LXR are activated by *O*-GlcNAc modification, either directly or indirectly. By contrast, FXR downregulates FAS transcription and it is suggested that *O*-GlcNAcylation of this nuclear receptor potentiates ChREBP activity and, accordingly, FAS expression. Overall, it appears that *O*-GlcNAcylation drives the transcription of FAS positively.

The reciprocal interplay between phosphorylation and *O*-GlcNAcylation modulates the properties of numerous proteins. FAS activity is regulated at the posttranslational level by AMPK-mediated phosphorylation; thus, the complex OGT–AMPK–FAS might exist but remains to be explored.

Fatty acid synthase and a small subset of OGT are resident in lipid microdomains. This common localization suggests that the two enzymes can be modified and, therefore, activated by tyrosine-phosphorylation. Also, a modification of FAS by OGT is not at all unlikely. Moreover, the production of lipid raft-specific fatty acids may contribute to the recruitment of OGT and speed up the sub-localization of the putative partners and the activation of mitogen signaling. Finally, as described for several proteins, *O*-GlcNAcylation of FAS could be protective against ubiquitin-dependent degradation, by promoting interaction with USP2a or by preventing interaction with COP1.

To date, the regulation of FAS activity and fate by *O*-GlcNAcylation, and even more so its crosstalk with OGT, have been neglected. Nevertheless, we consider this field of investigation of crucial importance since such a regulatory mechanism might confer a proliferative advantage on cells and induce tumor emergence.

## Author Contributions

SB and TL contributed equally to the writing of the manuscript.

## Conflict of Interest Statement

The authors declare that the research was conducted in the absence of any commercial or financial relationships that could be construed as a potential conflict of interest.
